# SERMs have substance-specific effects on bone, and these effects are mediated via ERαAF-1 in female mice

**DOI:** 10.1152/ajpendo.00488.2015

**Published:** 2016-04-05

**Authors:** Anna E. Börjesson, Helen H. Farman, Sofia Movérare-Skrtic, Cecilia Engdahl, Maria Cristina Antal, Antti Koskela, Juha Tuukkanen, Hans Carlsten, Andrée Krust, Pierre Chambon, Klara Sjögren, Marie K. Lagerquist, Sara H. Windahl, Claes Ohlsson

**Affiliations:** ^1^Rheumatology and Bone Diseases Unit, Centre for Genomic and Experimental Medicine, MRC Institute of Genetics and Molecular Medicine, Western General Hospital, University of Edinburgh, Edinburgh, United Kingdom;; ^2^Centre for Bone and Arthritis Research, Institute of Medicine, Sahlgrenska Academy, University of Gothenburg, Gothenburg, Sweden;; ^3^Strasbourg University, Faculté de Médecine, Institut d'Histologie, Strasbourg, France;; ^4^Department of Anatomy and Cell Biology, MRC Oulu, University of Oulu, Oulu, Finland;; ^5^Institut de Génétique et de Biologie Moléculaire et Cellulaire (Centre National de la Recherche Scientifique UMR7104; National de la Sante et de la Recherche Medicale U596; ULP, Collège de France), Illkirch, Strasbourg, France

**Keywords:** estrogen receptor, estrogen, selective estrogen receptor modulators, mouse, osteoporosis, activation function-1 of estrogen receptor-α

## Abstract

The bone-sparing effect of estrogens is mediated primarily via estrogen receptor (ER)α, which stimulates gene transcription through activation function (AF)-1 and AF-2. The role of ERαAF-1 for the estradiol (E_2_) effects is tissue specific. The selective ER modulators (SERMs) raloxifene (Ral), lasofoxifene (Las), and bazedoxifene (Bza) can be used to treat postmenopausal osteoporosis. They all reduce the risk for vertebral fractures, whereas Las and partly Bza, but not Ral, reduce the risk for nonvertebral fractures. Here, we have compared the tissue specificity of Ral, Las, and Bza and evaluated the role of ERαAF-1 for the effects of these SERMs, with an emphasis on bone parameters. We treated ovariectomized (OVX) wild-type (WT) mice and OVX mice lacking ERαAF-1 (ERαAF-1^0^) with E_2_, Ral, Las, or Bza. All three SERMs increased trabecular bone mass in the axial skeleton. In the appendicular skeleton, only Las increased the trabecular bone volume/tissue volume and trabecular number, whereas both Ral and Las increased the cortical bone thickness and strength. However, Ral also increased cortical porosity. The three SERMs had only a minor effect on uterine weight. Notably, all evaluated effects of these SERMs were absent in ovx ERαAF-1^0^ mice. In conclusion, all SERMs had similar effects on axial bone mass. However, the SERMs had slightly different effects on the appendicular skeleton since only Las increased the trabecular bone mass and only Ral increased the cortical porosity. Importantly, all SERM effects require a functional ERαAF-1 in female mice. These results could lead to development of more specific treatments for osteoporosis.

estrogens are major endocrine regulators involved in the skeletal growth and maintenance in both men and women (20, 28, 51). The bone-sparing effect of estrogens is mediated mainly via estrogen receptor (ER)α, but the effect of ERα can be slightly modulated by ERβ in female mice (37, 47, 54, 56). Although treatment with estrogens increases bones mass, it is associated with adverse effects such as an increased risk for venous thromboembolism and breast cancer (9, 14). Selective estrogen receptor modulators (SERMs) exert both agonistic and antagonistic effects in a tissue-specific manner by binding to the ERs (26). Some SERMs exert agonistic effects in bone and antagonistic effects in breast, but although they have less adverse effects than estradiol (E_2_), they still increase the risk for, e.g., venous thromboembolism (12, 13, 16, 46). Thus, it is of importance to further characterize the tissue-specific signaling pathways of SERMs to develop new bone-specific SERMs.

Recently, many studies have focused on identifying target cells for the estrogenic effects of ERα in bone (24, 52). These studies, together with studies on the importance of certain domains of ERα, have made the signaling pathways of ERα in bone clearer. It is now known that estrogen signaling via ERα in osteoblast lineage cells is crucial for the cortical bone mass in female mice (1, 23, 24, 29, 44, 52). For the trabecular bone mass in female mice, estrogen signaling via ERα in osteoclast lineage cells is important (25, 35, 44), but there is also moderate evidence that late osteoblast lineage cells are involved (23, 29, 44). In addition, we have demonstrated recently that ERαAF-2 seems to be required for all estrogenic effects in all tissues (6), whereas the role of ERαAF-1 for the effects of E_2_ in females is tissue specific, with a crucial role in trabecular bone and uterus but not in cortical bone or for vasculoprotective actions (5, 6). The signaling pathways of SERMs via ERα are not yet fully investigated in female mice.

In vitro studies have shown that the estrogen-induced transactivation of ERα is mediated by the ligand-independent activation function (AF)-1 in the NH_2_-terminal and/or the ligand-dependent AF-2 in the ligand-binding domain of ERα. It has been shown that the full ligand-dependent transcriptional activity of ERα is reached through a synergism between ERαAF-1 and ERαAF-2 (22, 30, 31, 38, 49). The AFs interact with several different coregulators (coactivators/corepressors). The balance of the coregulators is a critical determinant of the ability of ERα to regulate gene transcription, and this balance differs between cell types (4, 31, 49). Some coregulators are specific for either ERαAF-1 or ERαAF-2, whereas some coregulators bind to both (27). Variations in the expression of coregulators and the recruitment of coregulators to the ERα in different cell types also appear to have an important role for the tissue-specific effects of SERMs (4, 48). It is thus interesting to study the in vivo role of ERαAF-1 for the effects of SERMs in female mice.

In contrast to E_2_, many SERMs have a bulky side chain that protrudes from the ligand-binding pocket of ERα, which hinders the formation of ERαAF-2 (7). In vitro studies of the SERM-receptor complex suggest that the effects of SERMs involve ERαAF-1 and regions of ERα other than ERαAF-2 (4, 7, 17, 36, 40, 45, 48). However, we recently showed in vivo that female mice lacking ERαAF-2 (ERαAF-2^0^ mice) did not respond to SERM treatment in the estrogen-responsive tissues bone, uterus, and thymus (32), suggesting that a functional ERαAF-2 is also crucial for the ability of the SERMs to activate ERα.

Cortical bone mass is a major determinant of bone strength and nonvertebral fracture risk (59). In addition, the cortical microstructural parameter cortical porosity has recently emerged as a major determinant of cortical bone quality and thereby cortical bone strength. Importantly, cortical porosity has recently been described to predict fracture risk independently of areal bone mineral density (aBMD) (2, 3, 59). It has also been proposed that age-related increase in cortical porosity is a major determinant of age-dependent reduction of cortical bone strength (8, 43, 57, 58). The regulation of cortical porosity is mainly unknown, but we recently showed that serum E_2_ levels are inversely associated with cortical porosity (50). It is not reported whether SERMs regulate cortical porosity.

The three SERMs raloxifene (Ral), lasofoxifene (Las), and bazedoxifene (Bza) all reduce the risk for vertebral fractures (12, 13, 16, 46). In addition, Las, but not Ral, has been shown to reduce the risk for nonvertebral fractures in postmenopausal women (13, 16). Also, Bza has been shown to reduce the risk for nonvertebral fractures in women who have a higher risk for fractures (46). The aim of the present study was to compare the tissue specificity of Ral, Las, and Bza and evaluate the in vivo role of ERαAF-1 for the effects of these SERMs in ovariectomized (OVX) female mice, with an emphasis on axial and appendicular microstructural bone parameters.

## MATERIALS AND METHODS

### Animals

The Ethics Committee of the University of Gothenburg approved all experimental procedures involving animals (permit no. 251-2009). The mice had free access to fresh water and chow. Before surgery all animals received analgesics, and all surgery was performed under isoflurane inhalation anesthesia. All efforts were made to minimize suffering. The generation of ERαAF-1^0^ mice (5, 6) has been described previously. Briefly, the ERαAF-1^0^ mice have a specific deletion of AF-1 and do not express any full-length 66-kDa ERα protein. Instead, they express a truncated 49-kDa ERα protein that lacks AF-1 and also the physiologically occurring but less abundantly expressed 46-kDa ERα isoform. The ERαAF-1^0^ protein has been shown in vivo to be expressed in similar amounts as the WT ERα protein (5). The ERαAF-1^0^ mice and their littermate WT controls were inbred C57BL/6 mice and generated by breeding heterozygous females and males.

**Fig. 1.**
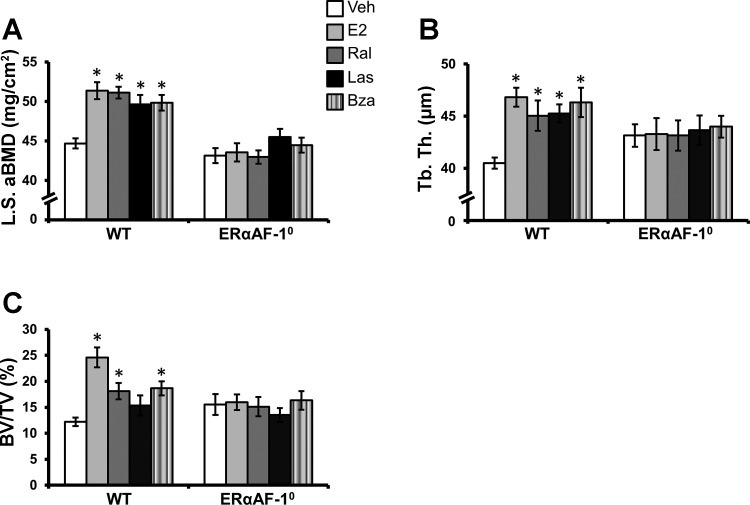
Effects of selective estrogen receptor modulator (SERM) treatment on the axial skeleton in ovariectomized (OVX) wild-type (WT) mice and OVX mice lacking activation function (AF)-1 of estrogen receptor-α (ERαAF-1^0^). OVX WT and ERαAF-1^0^ mice were treated with vehicle (Veh), estradiol (E_2_), raloxifene (Ral), lasofoxifene (Las), or bazedoxifene (Bza) for 3 wk. *A*: lumbar spine (LS) areal bone mineral density (aBMD) was analyzed by dual-energy X-ray absorptiometry. *B* and *C*: trabecular thickness (Tb.Th.) in the lumbar vertebra 5 (*B*) and trabecular bone volume/tissue volume (BV/TV; *C*) were analyzed by microcomputed tomography (μCT). *P < 0.05 vs. vehicle-treated OVX mice, Student's *t*-test Bonferroni corrected. Values are given as means ± SE (*n* = 7–11).

OVX was performed on 8-wk-old ERαAF-1^0^ and WT control mice. After 1 wk of recovery the OVX mice were weight matched into treatment groups receiving subcutaneous injections 5 days/wk for 3 wk with either vehicle (Veh; Miglyol 812; OmyaPeralta, Hamburg, Germany), E_2_ (1 μg·mouse^−1^·day^−1^; Sigma-Aldrich, St. Louis, MO), Ral (60 μg·mouse^−1^·day^−1^; Sigma-Aldrich), Las (4 μg·mouse^−1^·day^−1^; Pfizer, Groton, CT), or Bza (12 μg·mouse^−1^·day^−1^; Pfizer) (*n* = 7–11 mice/group). The mice were housed together according to treatment group. After 3 wk of treatment, the blood was collected from the axillary vein under anesthesia with ketamine (Pfizer, Sollentuna, Sweden) and dexdomitor vet (Orion, Espoo, Finland), and the mice were subsequently euthanized by cervical dislocation. The E_2_ and Ral doses were the same as in our previous experiments (15, 18), where the E_2_ dose has been shown to correspond to low diestrus E_2_ levels in mice (39). The Las and Bza doses chosen were based on previously published dose-response studies in rats (19, 21). We aimed for a dose that gave a clear effect on BMD in rats. To convert these doses to a similar dose in mice, body surface area calculations were performed (42). The doses used for Ral, Las, and Bza are shown relevant because of their ability to increase the lumbar spine aBMD to the same extent as E_2_ (Fig. 1*A*). Studies in rats have shown that the half-lives of these SERMs are 13.5 h for Ral (11), 9.4 h for Las (41), and 3.8 h for Bza (10). The substances were given once/day, 5 days/wk, for 3 wk. Since this is a long-term treatment and we are interested mainly in the static bone parameters, which respond slowly to changes, the small difference in half-life of these substances does probably not have an effect on these outcomes. The mice remained healthy throughout the experiment.

### Dual-Energy X-Ray Absorptiometry

Analyses of total body aBMD and lumbar spine aBMD were performed by dual-energy X-ray absorptiometry (DEXA) using the Lunar PIXImus mouse densitometer (Wipro GE Healthcare, Madison, WI).

### Microcomputed Tomography

Microcomputed tomography (μCT) analyses on the axial skeleton were performed on the lumbar vertebra 5 (L_5_) by using a Skyscan (Aartselaar, Belgium) 1072 scanner imaged with an X-ray tube voltage of 100 kV and current of 98 μA, with a 1-mm aluminum filter (34). The scanning angular rotation was 180° and the angular increment 0.90°. The voxel size was 6.51 μm isotropically. Data sets were reconstructed using a modified Feldkamp algorithm and segmented into binary images using adaptive local thresholding (53). The trabecular bone in the vertebral body caudal of the pedicles was selected for analyses, as described previously (6).

### High-Resolution μCT

High-resolution μCT analyses on the appendicular skeleton were performed on the distal femur by using an 1172 model μCT (Bruker Micro-CT, Aartselaar, Belgium). The femurs were imaged with an X-ray tube voltage of 50 kV and current of 201 μA, with a 0.5-mm aluminum filter. The scanning angular rotation was 180° and the angular increment 0.70°. The voxel size was 4.48 μm isotropically. The NRecon (version 1.6.9) was employed to perform the reconstruction following the scans (33). In the femur, cortical measurements were performed in the diaphyseal region of the femur starting at a distance of 4.89 mm from the growth plate and extending a further longitudinal distance of 449 μm in the proximal direction. For BMD analysis, the equipment was calibrated with ceramic standard samples. The porosity was evaluated for the outer 49.3 μm of the cortical bone to avoid endosteal trabecular bone to interfere with the analysis.

### Three-Point Bending

Immediately after the dissection, the femurs were fixed in Bürkhardt's formaldehyde for 2 days and after that stored in 70% ethanol. The bones were rinsed in PBS for 24 h before the mechanical testing. The three-point bending test (span length 5.5 mm, loading speed 0.155 mm/min) at the midfemur was made using an Instron universal testing machine (Instron 3366; Instron, Norwood, MA). Based on the recorded load deformation curves, the biomechanical parameters were acquired from raw files produced by Bluehill 2 software version 2.6 (Instron) with custom-made Excel macros.

### Statistical Analysis

For statistical evaluation, Student's *t*-test with Bonferroni correction was used for comparing the E_2_-, Ral-, Las-, and Bza-treated groups with the Veh-treated group (4 comparisons).

## RESULTS

### The Skeletal Effects of SERMs in WT Mice

**Fig. 2.**
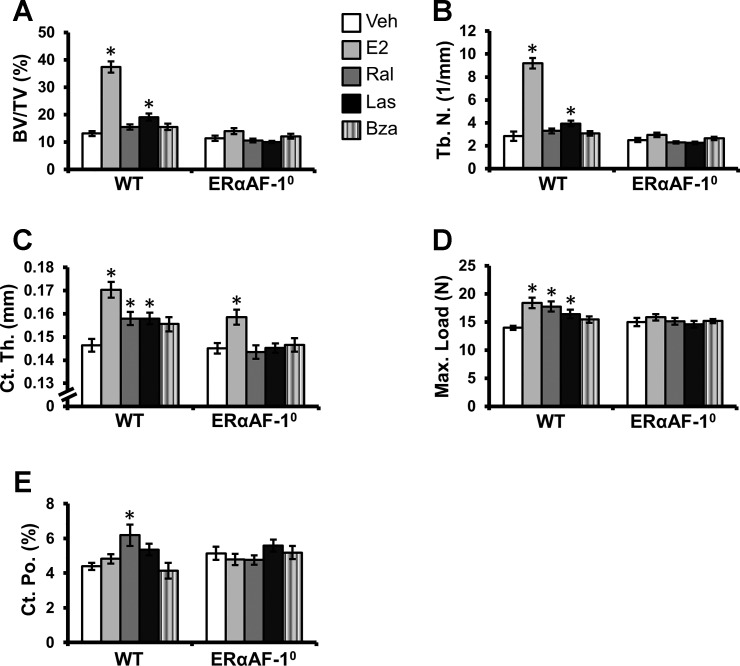
Effects of SERM treatment on the appendicular skeleton in OVX WT and ERαAF-1^0^ mice. OVX WT and ERαAF-1^0^ mice were treated with vehicle (Veh), estradiol (E_2_), raloxifene (Ral), lasofoxifene (Las), or bazedoxifene (Bza) for 3 wk. Trabecular bone volume/tissue volume (BV/TV; *A*), trabecular number (Tb. N.; *B*), and cortical thickness (Ct. Th; *C*) in the femur were analyzed by high-resolution μCT. Maximal load at failure (Max.Load) of the femur was analyzed by 3-point bending (*D*), and femoral cortical porosity (Ct. Po) was analyzed by high-resolution μCT (*E*). *P < 0.05 vs. Veh-treated OVX mice, Student's *t*-test Bonferroni corrected. Values are given as means ± SE (*n* = 7–10).

OVX WT mice were treated with three different SERMs (Ral, Las, or Bza), E_2_, or Veh to evaluate the effects of these SERMs on E_2_-responsive bone parameters. All evaluated bone parameters in the axial (Fig. 1) and appendicular (Fig. 2) skeleton (except for cortical porosity) had a clear response to E_2_ treatment. The effects of SERMs on the axial and appendicular skeleton are presented below. DEXA analysis showed that total body aBMD was increased by all three SERMs compared with Veh-treated mice (Table 1).

**Table 1.** Effects of SERM treatment in OVX WT and ERαAF-1^0^ mice

	OVX WT					Ovx ERαAF-1^0^				
	Veh	E_2_	Ral	Las	Bza	Veh	E_2_	Ral	Las	Bza
Total body aBMD, mg/cm^2^	41.5 ± 0.4	46.0 ± 0.8*	44.4 ± 0.5*	43.6 ± 0.6*	43.9 ± 0.3*	40.8 ± 0.5	42.4 ± 0.7	41.1 ± 0.4	41.0 ± 0.6	41.6 ± 0.4
Uterus weight/BW, %	0.045 ± 0.002	0.582 ± 0.038*	0.083 ± 0.004*	0.151 ± 0.006*	0.057 ± 0.005	0.025 ± 0.001	0.121 ± 0.009*	0.029 ± 0.003	0.027 ± 0.002	0.025 ± 0.003
Thymus weight/BW, %	0.33 ± 0.012	0.096 ± 0.006*	0.27 ± 0.011*	0.27 ± 0.010*	0.29 ± 0.013	0.32 ± 0.015	0.31 ± 0.011	0.33 ± 0.015	0.29 ± 0.010	0.31 ± 0.016

SERM, selective estrogen receptor modulator; OVX, ovariectomized; WT, wild type; ERαAF-1^0^, OVX mice lacking activation function (AF)-1 of estrogen receptor-α; Veh, vehicle; E_2_, estradiol; Ral, raloxifene; Las, lasofoxifene; Bza, bazedoxifene; aBMD, areal bone mineral density; BW, body weight.

OVX WT and ERαAF-1^0^ mice were treated with Veh, E_2_, Ral, Las, or Bza for 3 wk. The total body aBMD was analyzed by dual-energy X-ray absorptiometry.

* *P* < 0.05 vs. Veh-treated OVX mice, Student's t-test Bonferroni corrected. Values are given as means ± SE (*n* = 7–11).

#### Axial skeleton.

The axial skeleton was specifically analyzed by DEXA and μCT. All three SERMs increased the lumbar spine aBMD, and their effects were comparable with the effect of E_2_ (Fig. 1*A*). Further analysis of the axial skeleton showed that all three SERMs increased the trabecular thickness in the vertebrae (Fig. 1*B*). Ral and Bza treatment also increased the vertebral trabecular bone volume/tissue volume (BV/TV), and a similar nonsignificant trend was seen in the Las-treated mice (Fig. 1*C*).

#### Appendicular skeleton.

The appendicular skeleton was analyzed by high-resolution μCT. This showed that Las was the only SERM increasing the trabecular BV/TV (Fig. 2*A*) and trabecular number (Tb.N.; Fig. 2*B*) in the distal metaphyseal region of the femur, albeit not to the same extent as E_2_ (E_2_: 186% increase in BV/TV and 224% increase in Tb.N.; Las: 46% increase in BV/TV and 39% increase in Tb.N.). The cortical thickness in the middiaphyseal region of femur was increased when the mice were treated with Ral and Las (Fig. 2*C*) compared with Veh-treated controls. For the Bza-treated mice, the increase in cortical thickness compared with Veh-treated controls did not reach significance after the conservative Bonferroni correction (*P* = 0.023, for 4 comparisons Bonferroni correction requires *P* < 0.013; Fig. 2*C*). In accord with the increase in cortical thickness, the biomechanical strength of the middiaphyseal region of femurs was increased after treatment with Ral and Las (Fig. 2*D*). Interestingly, however, Ral, but not Las or Bza, increased the cortical porosity (Fig. 2*E*).

### The Effects of SERMs in Uterus and Thymus in WT Mice

The effects of Ral, Las, and Bza were also evaluated in the major estrogen target tissues: uterus and thymus. As expected, E_2_ increased the uterine weight, whereas it reduced the thymus weight in the OVX WT mice. The three SERMs exerted no or minor estrogenic effects on uterine weight (Table 1). The thymus weight was slightly but significantly decreased in the OVX WT mice treated with Ral and Las but not in the OVX WT mice treated with Bza (Table 1).

### The Effects of SERMs Require a Functional ERαAF-1 in All Evaluated Tissues

Similarly, as we have shown recently (6), E_2_ exerted tissue-specific estrogenic effects in female ERαAF-1^0^ mice. Because in vitro studies indicate that ERαAF-1 is involved in mediating the estrogenic effects of SERMs, we evaluated the effect of Ral, Las, and Bza in OVX female ERαAF-1^0^ mice. Notably, there were no effects of Ral, Las, or Bza on any of the evaluated skeletal parameters, uterine, or thymus weight in the OVX ERαAF-1^0^ mice (Figs. 1 and 2 and Table 1). These results demonstrate that a functional ERαAF-1 is required for mediating the effects of Ral, Las, and Bza in all evaluated tissues.

## DISCUSSION

Estrogens and SERMs increase bone mass but also lead to severe adverse effects (9, 14). Thus, it is of importance to further characterize the tissue-specific signaling pathways of estrogens and SERMs. We herein compared the tissue specificity of Ral, Las, and Bza and evaluated the in vivo role of ERαAF-1 for the effects of these SERMs in OVX female mice, with a special emphasis on the microstructural parameters in axial and appendicular bone. We demonstrate that all three SERMs increase the trabecular bone mass in the axial skeleton and cortical thickness in the appendicular skeleton, although the Bza-treated group is borderline significant. We speculate that there is a possibility that the quality of the metaphyseal region of the appendicular skeleton is better in the Las-treated group than in the Ral-treated group, shown by a significantly increased femoral trabecular bone mass in the Las-treated group. In addition, the Ral-treated group had an increased femoral cortical porosity that suggests reduced cortical bone quality. However, the results from the maximal load at failure in the diaphyseal region of the femur oppose this conclusion since the Ral-treated mice had bones at least as strong as the Las-treated mice. Importantly, all evaluated bone and nonbone effects of these SERMs required a functional ERαAF-1.

It is well established that the SERM Ral reduces the risk for vertebral but not nonvertebral fractures in postmenopausal women (16). The strength of the axial skeleton is highly dependent on the trabecular bone, whereas appendicular skeleton has a higher cortical bone content. One may speculate that Ral exerts mainly beneficial effects on axial trabecular bone. The newer SERMs Las and, to some extent, also Bza reduce the risk of both vertebral and nonvertebral fractures (13, 46). Therefore, it is possible that Las and Bza would exert an overall better effect on both cortical and trabecular bone of the appendicular skeleton. We demonstrated that Ral, Las, and Bza all increased the trabecular bone mass in the axial skeleton, shown by increased lumbar spine aBMD and trabecular thickness in vertebra. This is consistent with the fact that they all reduce vertebral fracture risk in humans (13, 16, 46). Both Ral and Las significantly increased the cortical bone thickness in the appendicular skeleton, whereas the effect of Bza was only nominally significant. The increase in cortical thickness correlates well with the bone strength, evaluated by three-point bending, since both Ral and Las had an increased maximal load at failure. Interestingly, a possible explanation for the lack of significant effect of Ral on nonvertebral fracture risk could be due to the finding that Ral significantly increased cortical porosity in the appendicular skeleton. Thus, the stimulatory effect of Ral on cortical bone mass may be somewhat counteracted by the increased cortical porosity. In contrast, Las (and Bza) increased the cortical thickness without significantly increasing the cortical porosity. It is also worth noticing that it was only Las treatment that led to an increased trabecular BV/TV and trabecular number in the appendicular skeleton. Since the measurement of bone strength was made in the midfemur, where there is no trabecular bone, it is possible that the increased appendicular trabecular bone mass may also explain the effect of Las on nonvertebral fractures since the proximal femur is a common site for osteoporotic fractures and consists of both cortical and trabecular bone. Collectively, we speculate that the findings of the effects of Ral and Las on axial and appendicular bone microstructure may at least partly explain why Ral and Las reduce the risk for vertebral fractures, whereas Las, but not Ral, reduces the risk for nonvertebral fractures in humans.

In our previous study (6), we showed that the mice lacking AF-1 (ERαAF-1^0^) have an essentially normal E_2_ response on cortical bone thickness, i.e., 94 ± 12% of the E_2_ response in WT mice. In the present study, we show that E_2_ also give a clear E_2_ response in cortical bone. However, this time the response is less pronounced (47 ± 11% of the E_2_ response in WT mice). This might be explained by the different doses of E_2_, length of treatment, and routes of administration. In our previous study, the mice had a subcutaneous pellet inserted that continuously released 0.167 μg E_2_/day for 4 wk, whereas in this study the mice were injected with 1 μg E_2_/day, 5 days/wk, for 3 wk.

It is important to find the parts of ERα that are crucial for mediating the estrogenic responses from E_2_ or SERMs to find more specific treatments for postmenopausal osteoporosis. It has already been shown by using mouse models that different parts of ERα may be more or less important for mediating the estrogenic effects in different tissues (5, 6, 32, 55). We have demonstrated recently that the role of ERαAF-1 for the effects of E_2_ in female mice is tissue specific (6), but the in vivo role of ERαAF-1 for the effects of SERMs in female mice is unknown. In vitro studies have suggested that ERαAF-1 is the most important AF for mediating the SERM effects (4, 7, 17, 36, 40, 45, 48). The results in this study show, for the first time in vivo in females, that ERαAF-1 is required for mediating the effects of Las, Ral, and Bza on all evaluated bone parameters, uterus, and thymus. In addition, we have reported recently that the ERαAF-2 is required for mediating the bone, uterus, and thymus effects of SERMs (32). Together, these studies suggest that both a functional ERαAF-1 and ERαAF-2 are crucial for the SERMs to have an effect. Since it has been shown that helix 12 interacts with the static part of ERαAF-2 and mimics a coregulatory binding when a SERM is bound (7, 45), we speculate that when the ERα amino acids 543–549 in helix 12 are deleted (as in ERαAF-2^0^ mice), helix 12 cannot interact with this static ERαAF-2 region when a SERM is bound. The static part would then still be visible and possibly be able to interact with corepressors, repressing the activity of the SERMs, which now would not be able to activate ERα via ERαAF-1 (7, 36, 45). In addition, if ERαAF-1 is deleted and a SERM binds to the ERα, the helix 12 interacts with the static part of ERαAF-2, and no coregulators can interact with either ERαAF-1 or ERαAF-2. The ERα-dependent gene transcription would thus probably be absent in the evaluated tissues in the ERαAF-1^0^ mice (4, 17, 40, 48). We speculate that this is the reason why both a functional ERαAF-1 and ERαAF-2 are required for mediating the effects of SERMs. In addition, the SERM-ERβ interactions are not able to replace ERα in the evaluated tissues in the female mice.

One of the most important characteristics of all SERMs is that they have fewer agonistic effects on the reproductive system than E_2_ (26). Our results are in accord with this since we show that none of the SERMs had a major estrogenic response in the uterus, and although Ral and Las increase the uterus weight significantly, their response is less than 20% of the E_2_ response.

In conclusion, all three SERMs increase the axial trabecular bone mass. Ral and Las increase appendicular cortical thickness, but Ral also increases cortical porosity, whereas Las increases the appendicular trabecular bone mass. These findings may explain why all three SERMs reduce the risk for vertebral fractures, whereas Las, but not Ral, reduces the risk for nonvertebral fractures. Our data suggest that SERMs can regulate cortical porosity; however, the mechanism for this needs to be evaluated further. Importantly, all of the effects of Ral, Las, and Bza activate ERα via the ERαAF-1 in female mice, whereas E_2_ can activate the ERα in cortical bone via parts of the ERα other than ERαAF-1. It may be favorable to develop a new class of SERMs for treatment of female osteoporosis, not acting via the ERαAF-1, to have beneficial effects on nonvertebral fracture risk without having major adverse effects on the reproductive system in female mice.

## GRANTS

This study was supported by the Swedish Research Council, COMBINE, an ALF/LUA research grant in Gothenburg, Sweden, the Lundberg Foundation, the Torsten and Ragnar Söderberg's Foundation, the Novo Nordisk Foundation, the King Gustav V's 80 Years' Foundation, the Association Against Rheumatism, the Åke Wiberg Foundation, and National Institute of Diabetes and Digestive and Kidney Diseases Grant DK-071122.

## DISCLOSURES

The authors have nothing to disclose.

## AUTHOR CONTRIBUTIONS

A.E.B., P.C., and C.O. conception and design of research; A.E.B., H.H.F., S.M.-S., C.E., M.C.A., A. Koskela, J.T., A. Krust, K.S., M.K.L., and S.H.W. performed experiments; A.E.B., H.H.F., and C.O. analyzed data; A.E.B., S.M.-S., H.C., M.K.L., S.H.W., and C.O. interpreted results of experiments; A.E.B. prepared figures; A.E.B. drafted manuscript; A.E.B. and C.O. edited and revised manuscript; A.E.B., S.M.-S., C.E., M.C.A., A. Koskela, J.T., H.C., A. Krust, P.C., K.S., M.K.L., S.H.W., and C.O. approved final version of manuscript.
